# No innocent bystanders: pertussis vaccination and evolutionary parallelisms between Bordetella parapertussis and Bordetella pertussis

**DOI:** 10.1099/mgen.0.001544

**Published:** 2025-11-14

**Authors:** Valérie Bouchez, Albert Moreno-Mingorance, Alba Mir-Cros, Annie Landier, Nathalie Armatys, Sophie Guillot, Maria Teresa Martín-Gómez, Carla Rodrigues, Julie Toubiana, Ana I. Bento, Michael R. Weigand, Juan José González-López, Sylvain Brisse

**Affiliations:** 1 Biodiversity and Epidemiology of Bacterial Pathogens, Institut Pasteur, Université Paris Cité, Paris, France; 2 National Reference Center for Whooping Cough and Other Bordetella Infections, Institut Pasteur, Paris, France; 3 Vall d'Hebron Institut de Recerca (VHIR), Vall d'Hebron Barcelona Hospital Campus, Barcelona, Spain; 4 CIBER de Enfermedades Infecciosas (CIBERINFEC), Instituto de Salud Carlos III, Madrid, Spain; 5 Department of Clinical Microbiology, Hospital Universitari Vall d'Hebron, Barcelona, Spain; 6 Department of General Pediatrics and Pediatric Infectious Diseases, Hôpital Necker–Enfants Malades, APHP, Université Paris Cité, Paris, France; 7 Department of Public & Ecosystem Health, College of Veterinary Medicine, Cornell University, Ithaca, NY, USA; 8 Division of Bacterial Diseases, Centers for Disease Control and Prevention, Atlanta,, Georgia, USA; 9 Department of Genetics and Microbiology, Universitat Autònoma de Barcelona, Barcelona, Spain

**Keywords:** *Bordetella parapertussis*, phylogenetic diversity, vaccine driven evolution, virulence factors

## Abstract

Pathogens adapting to the human host and to vaccination-induced immunity may follow parallel evolutionary paths. *Bordetella parapertussis* (*Bpp*) contributes significantly to the burden of whooping cough (pertussis) and shares vaccine antigens with *Bordetella pertussis (Bp)*; both pathogens are phylogenetically related and ecological competitors. *Bp* vaccine antigen-coding genes have accumulated variation, including pertactin (PRN) disruptions, after the introduction of acellular vaccines in the 1990s. We aimed to evaluate evolutionary parallelisms in *Bpp*, even though pertussis vaccines were designed against *Bp*. We sequenced 242 *Bpp* isolates collected in France, the USA and Spain between 1937 and 2019, spanning pre-vaccine and two vaccines eras. We investigated the temporal evolution of *Bpp* sublineages using a Bayesian approach based on whole-genome SNPs and performed comparative genomic analyses focusing on antigen and virulence gene loci. The most recent common ancestor of all sequenced *Bpp* isolates was estimated around the year 1877, making it one of the youngest human pathogens, and the *Bpp* evolutionary rate we estimated (2.12×10^−7^ substitutions per site per year) was remarkably similar to the one previously reported for *Bp* (2.24×10^−7^). PRN antigen deficiency in *Bpp* was driven by 18 disruptive mutations, including deletion *prn*:ΔG-1895 estimated to have occurred around 1998 and observed in 73.8 % (149/202) of post-2007 *Bpp* isolates. In addition, we detected two early (year ~1900) mutations in the *bvg*A-*fhaB* intergenic region, which controls multiple virulence factors including the filamentous haemagglutinin antigen. Gene clusters for pertussis toxin and fimbriae showed a surprising lack of gene decay. Our findings suggest early adaptation of *Bpp* to humans through modulation of the *bvgAS* regulon, and a rapid adaptation through the loss of PRN expression, representing a late evolutionary parallelism concomitant with acellular vaccination against whooping cough.

**Impact Statement**

Vaccination against *Bordetella pertussis* (*Bp*) has strongly affected the recent evolution of this main agent of whooping cough. Whether it may have done so co-incidentally on *Bordetella parapertussis* (*Bpp*)*,* which is genetically and ecologically very similar to *Bp,* has not been described in detail. Our findings show striking evolutionary parallelisms of *Bpp* with *Bp*, including early changes in a critical regulatory region of virulence, and strong evidence of adaptation to vaccine-driven population immunity, even though whooping cough vaccines were not designed explicitly against *Bpp*. The rapid populational loss of pertactin in countries where acellular pertussis vaccines are used may also reduce protection by vaccination against *Bpp*, the second agent of whooping cough.

## Data Summary

1. The genome sequence reads of the 250 *Bordetella parapertussis* (*Bpp*) strains were deposited in the European Nucleotide Archive at https://www.ebi.ac.uk under projects PRJEB45017, PRJEB5039, PRJEB29316, PRJEB2850 and PRJEB2274, or in the National Center for Biotechnology Information under project numbers PRJNA731630, PRJNA287884 and PRJDB7578 (Table S1).
2. A supplementary Appendix also includes: (1) Supplementary methods: Microbiological characterization, DNA preparation and genomic sequencing, Phylogenetic dynamics; (2) Supplementary results: Characteristics of four main *Bpp* lineages/sublineages, SNP densities per functional category and Bvg label, Homoplasic SNPs, Variation in genes associated with lipopolysaccharide, toxins and autotransporters, Genomic structure and rearrangements, Insertion sequences; (3) Supplementary Tables: Table S1**:** Characteristics of isolates, Table S2: SNPs identified in *Bpp,* Table S3: SNP densities according to functional categories and *bvg* status, Table S4: Insertions and deletions detected by Snippy in all samples against BPP12822, Table S5: Pertactin sequence variants observed in *Bpp* isolates; (4) Supplementary Figures: Fig. S1. Root-to-tip genetic divergence versus time, Fig. S2. Effective population size analysis, Fig. S3. Mutations within the *bvgA-fhaB* intergenic sequences of *Bordetella pertussis* (*Bp*)*,* Fig. S4. *Bp* and *Bpp* isolates collected in France, per year (1996–2019), Fig. S5. SNP densities according to functional categories (panel A) and to bvg-regulation (panel B), Fig. S6. *ptxP* promoter sequence in *Bpp,* Fig. S7. Correspondence between PFGE groups and genomic lineages/sublineages/clades in *Bpp;* and (5) Supplementary references.

## Introduction

Public health interventions aimed at controlling specific pathogens may concomitantly affect non-target human commensal or pathogenic organisms. For example, commensal bacteria may evolve antimicrobial resistance in response to antimicrobial therapy against pathogens, a phenomenon called bystander evolution [1] that has far-reaching implications in microbial ecology and public health [2]. So far, there is little or no evidence of bystander evolution under vaccination-induced immune pressure [3].

Whooping cough (pertussis) is a human respiratory disease caused mainly by *Bordetella pertussis (Bp*). In 2014, globally, >24 million pertussis cases causing >160,000 deaths in children under 5 years of age were estimated [4]. Like *Bp*, *Bordetella parapertussis (Bpp*) causes whooping cough, though the disease is typically less severe [5,7] and thus reported much less frequently than *Bp* infections [8,9]. Furthermore, *Bpp* infection is not reportable in many countries, including the USA. The first whole-cell pertussis vaccines (wPVs) were already developed using *Bp* strains when *Bpp* was first reported in 1938 [5]. *Bpp* is still not considered as a target of vaccines against whooping cough, which are designed only from *Bp* antigens. Although *Bpp* was initially considered as an obligate human pathogen, in 1987 a few *Bpp* isolates were reported in lambs [10]. As sheep *Bpp* isolates form a distinct lineage from human *Bpp* isolates, they are not considered further herein.

While wPVs, produced using *Bp* strains, remain in use in most of the global South, acellular pertussis vaccines (aPVs) were adopted in the mid-1990s and 2000s by many high-income countries. aPVs contain one to five *Bp* antigens: pertussis toxin (PT), which is always present, combined in most vaccines with filamentous haemagglutinin (FHA), pertactin (PRN) and/or type 2 and type 3 fimbriae (FIM2 and FIM3). It is well established that circulating *Bp* populations, which are human-restricted (as is *Bpp*), have evolved in response to vaccine-induced immunity. For example, rates of evolution of vaccine antigen-encoding genes have accelerated since the introduction of aPV, compared to other surface protein genes [11]. Non-synonymous mutations (nsSNP) in PT, PRN, FHA, FIM2 and FIM3 encoding genes, as well as the promoter region of the PT gene cluster, have occurred and raised in frequency, often to fixation in extant *Bp* populations, compared to the pre-vaccine era [12]. Moreover, the rapid emergence of PRN-deficient *Bp* isolates has been observed in countries where aPV vaccination has been implemented [13,14], resulting from multiple independent mutation events rather than the spread of a few genotypes. PRN deficiency has reached near-fixation in early aPV-using countries [12,15], and confers a selective advantage during infection [16,17] with a higher fitness of PRN-negative isolates in the aPV era [18]. The vaccine-driven evolution of *Bp* is regarded as a prominent example of global population-level effects of large-scale vaccination [19,20].

*Bpp* is closely related to *Bp*. Of the five *Bp* vaccine antigens, *Bpp* expresses orthologs of PRN and FHA, but not PT and FIM2 and FIM3 proteins. PRN and FHA proteins are 91.5 and 95.2% identical in their amino acid sequence to their *Bp* counterparts, respectively [21,22]. Given that *Bpp* is phylogenetically related and antigenically similar to *Bp,* the possibility exists that *Bpp* may have also evolved under immune pressure exerted by vaccination targeting *Bp*. However, the effectiveness of pertussis vaccines in protecting children against *Bpp* infection is usually controverted [23]. PRN-deficient *Bpp* isolates have been observed since 2007 in France [24], but the emergence of PRN-deficient *Bpp* is otherwise undescribed.

*Bp* and *Bpp* have converged in adapting to their human-restricted niche independently, both having evolved from the genetically broader species *Bordetella bronchiseptica*, an ecological generalist observed in multiple animal host species [25,26]. Among other events, the evolution of *Bp* has involved gene loss, genomic rearrangements and mutations in the intergenic region between the genes coding for FHA and the BvgAS two-component master regulator of virulence [12,27].

Currently, little is known about the evolution of *Bpp*, largely because it has been rarely isolated in culture. The aim of this study was, by gathering a large international collection of human clinical isolates *of Bpp*, to define its population structure and evolution and explore whether signatures of evolution may be driven by pertussis vaccination-induced immunity.

## Methods

### Collection of *Bpp* clinical isolates

We collected a large biological resource dataset of *Bpp* isolates from three countries, considering all isolates available in the collection of their respective reference laboratory. In France, 119 *Bpp* isolates were collected at the National Reference Center for Whooping Cough and Other Bordetella Infections, isolated between 1958 and 2019. Most of these isolates were collected through the hospital-based paediatric network RENACOQ, which has operated continuously since 1996 [28,29] and which is still active and represents about one third of the country. From the USA, 85 *Bpp* isolates were collected by the Centers for Disease Control and Prevention (CDC), being gathered through routine surveillance and during outbreaks between 1937 and 2017, many of which were sequenced as part of a previous study [27]. From Spain, 38 isolates were collected from patients attending the hospitals and primary health care centres according to usual routine diagnostic procedures between 1993 and 2019 in three Spanish regions (Catalonia, Community of Madrid and Castilla-La Mancha). In addition, eight publicly available genomes of isolates originating from other countries (Australia, Japan, UK and Germany) were included. Details about the isolates' characteristics are provided in Table S1.

### Microbiological characterization, DNA preparation and genomic sequencing

Isolates were grown on Bordet-Gengou agar, antigen characterization was done by Western blot or ELISA, and DNA preparation and genomic sequencing were performed using Illumina technology; details are provided in the Supplementary Appendix (section Methods: Microbiological characterization, DNA preparation and genomic sequencing).

### Phylogenetic and gene content analyses

**Fig. 1.**
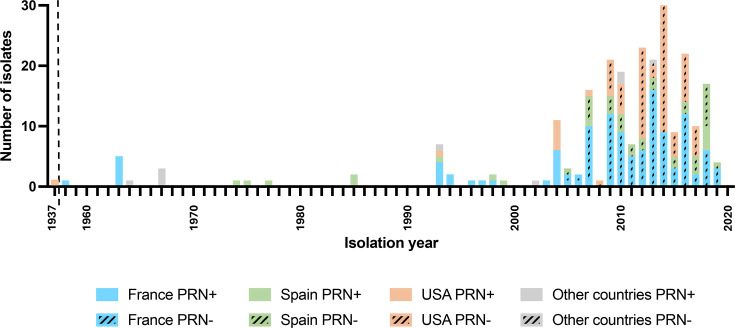
Number of *Bpp* isolates collected per year according to country of origin. The figure includes 242 study isolates and 8 isolates for which genomic sequences were publicly available. The data are broken down per year, except for the first bar. Blue: France; Green: Spain; Orange: USA; Grey: other countries (for publicly available genome sequences). Hatching represents PRN− isolates (as verified experimentally for France and Spain; and as deduced from genomic sequences for the USA and public sequences from other countries).

Raw reads were trimmed with Trimmomatic (v. 0.38). Snippy (v. 4.3.6) was used for SNP analysis with *Bpp* strain 12822 (GenBank accession no. BX470249.1) used as reference, as previously described [30], without removing recombinant or repetitive regions, as previously done by Safarchi *et al*. [31] and Fu *et al*. [32]. Indeed, the recombination analysis detected only a very small number of events, so the results were considered more stable and reliable when recombinant regions were not excluded. A maximum likelihood analysis based on the whole-genome SNPs was carried out with IQ-TREE (v. 1.6.10) using 1,000 bootstrap replicates. The existence of a temporal signal in the genomic data was estimated with TempEst by a regression analysis between the root-to-tip divergence in the maximum likelihood tree and the isolation year (Fig. 1). The software tool BEAST version 1.10.4 [33] was used to infer the phylogenetic dynamics as detailed in the Supplementary Appendix (section Methods: Effective population size dynamics).

We looked for transposases using ISMapper [34] or blastN (https://blast.ncbi.nlm.nih.gov/Blast.cgi) with IS*1001* (BPP0078) and IS*1002* (BPP1897) sequences as queries. We estimated the pan-genome using Roary (v. 3.13.0) [35] with default parameters (core genes defined as being present in 95% genomes; without paralogs) from gff3 archives previously annotated with bakta (v. 1.2.1) [36]. We looked for plasmids using PlasmidFinder (v. 2.1.1) [37].

### Mutations analysis and genotyping

We performed *prn* gene sequence analysis using blastN (https://blast.ncbi.nlm.nih.gov/Blast.cgi) with *Bpp* strain 12822 (NC_002928) gene sequence as queries. Genotyping of virulence genes was done using the BIGSdb platform (https://bigsdb.pasteur.fr/bordetella/) using *de novo* assemblies as previously detailed [38,39].

## Results

### *Bpp* genomic evolution, population structure and time-resolved phylogeny

We collected 242 *Bpp* isolates between 1937 and 2019 in France, the USA and Spain, conducted genomic sequencing and analysed the data using phylogenetic and population biology approaches. The number of isolates varies temporally, with a maximum of 32 isolates collected in 2014 (Fig. 1). Together with the eight additional public genome sequences, a dataset of 250 genomic sequences was analysed.

The average genome size was 4,732,038 bp and the average G+C % was 68.1 % (vs around 4.1 Mb and 67.7 G+C % for *Bp* [40]). No plasmid replicons were identified. Using ISMapper or blastN, the copy numbers of IS*1001* and IS*1002*, two mobile elements used for diagnosis of *Bpp* [40], were investigated in the 250 genomic sequences and successfully determined in 244 of these. We found 22 copies of IS*1001* and 9 copies of IS*1002* in, respectively, 225 and 231 assemblies. Only a few assemblies had different copy numbers of IS*1001* and IS*1002* (Table S1).

Gene content was highly conserved among the collection of isolates, indicating minimal gene gain or loss. In total, 5,329 different protein-coding genes were identified. Among these, 3,640 were present in at least 99% of isolates, and 4,269 in at least 95%.

**Fig. 2.**
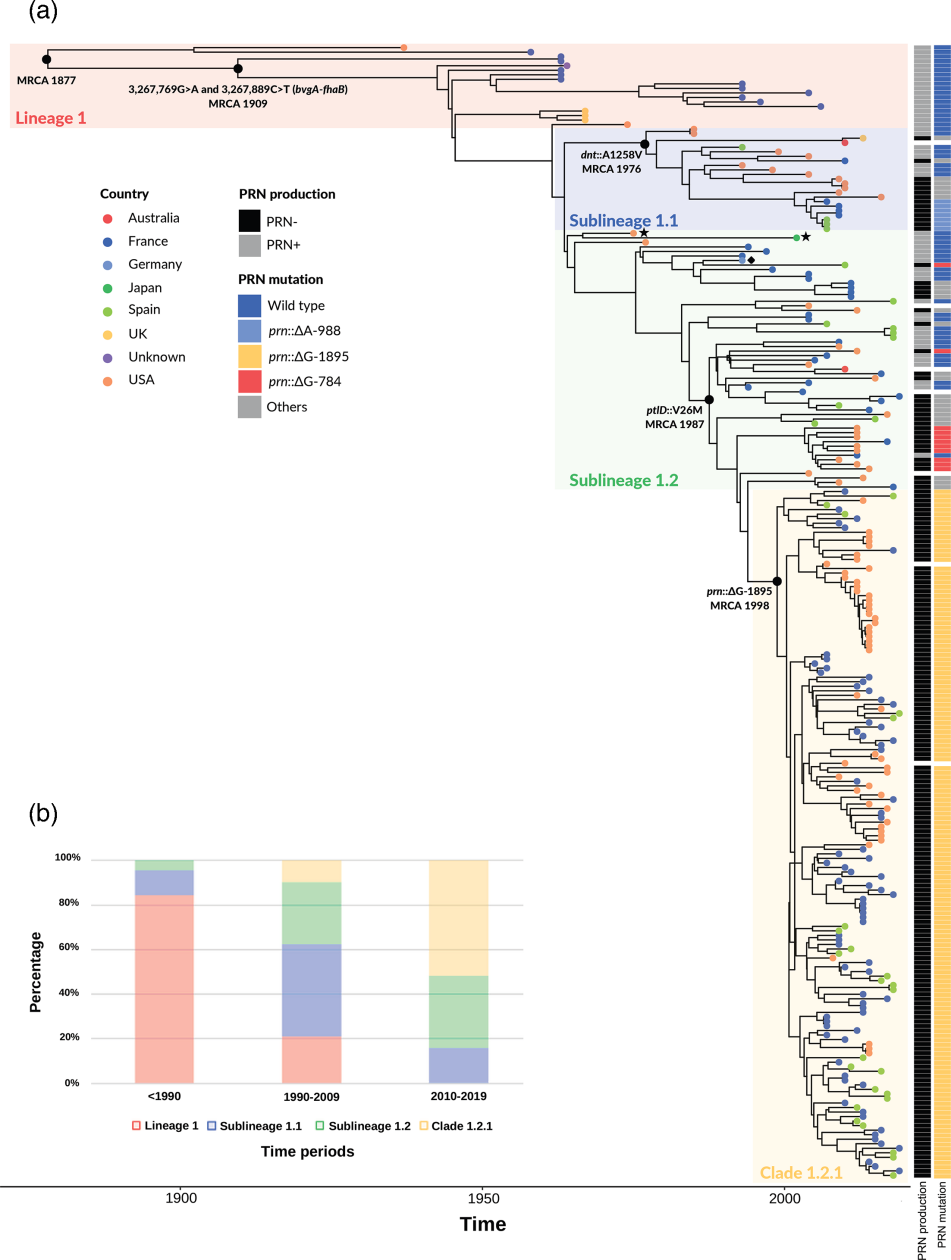
Time-scaled phylogeny of *Bpp*. Panel (a): Bayesian phylogenetic reconstruction of 250 *Bpp* isolates collected between 1937 and 2019. The phylogenetic tree was built using BEAST (strict clock and Bayesian Skygrid model) from whole-genome SNPs (compared to the reference strain 12822, GenBank accession BX470249.1). The reference strain belongs to sublineage 1.2 and its position is indicated by a black rhombus symbol. The two black stars indicate the two sublineage 1.2 isolates with the N142S change (see Table 1 footnote). The country of origin of the isolates is represented with coloured circles located at the tree leaves. PRN production status, with the three most frequent (>2 %) *prn* mutations associated with PRN deficiency, is indicated by the two columns on the right of the tree leaves (see colour key; see Table S1 for complete information; missing data are represented in white). Panel (b): proportions of *Bpp* phylogenetic groups according to three time periods (before 1990, 1990–2009 and 2010–2019).

We next investigated the genomic evolution of *Bpp*. Genome-wide nucleotide variation analysis identified 1,994 SNPs (Table S2), which were used for phylogenetic analysis (Fig. 2a). On average, strains differed by 47 pairwise SNPs (ranging from 1 to 214). Of the 1,994 SNPs, 35.7% were phylogenetically informative, i.e. the variation was observed in at least two genomes. A strong temporal signal of SNP accumulation over time was found, with a root-to-tip genetic divergence versus time of isolation regression parameter R^2^=0.85 (Fig. S1). Using Bayesian analysis, we estimated the mean evolutionary rate of *Bpp* as 2.1×10^−7^ substitutions per site per year (95% highest posterior density [HPD]: 1.9×10^−7^, 2.3×10^−7^ substitutions per site per year), corresponding to 1.02 substitutions per genome per year. The most recent common ancestor (MRCA) of our *Bpp* dataset was estimated in 1877 (95 % HPD: 1865 to 1889). Using this SNP variation, we estimated the fluctuation of the effective size of the *Bpp* population reflected by our dataset since 1960 (see details in the Supplementary Appendix, section: Results, Effective population size analysis).

Phylogenetic reconstruction (Fig. 2a) uncovered a scaled population structure, within which four main phylogenetic groups were defined (summarized in Table 1; see also Characteristics of four main *Bpp* phylogenetic groups in the Supplementary Appendix). The sampling frequency of these lineages varied with time (Fig. 2b).

**Table 1.** Main characteristics of the four *Bpp* phylogenetic groups

Lineage/sublineage/clade	Total no. of isolates	<**1990**	1990–**2009**	2010–**2019**	Genetic markers	% Pertactin deficient (no./total)	Correspondence with clades in Safarchi *et al.* current microbiol. 2022
**1**	18	12	6	0	BPP_RS11415-N142S	0 % (0/18)	Clade 1
**1.1**	23	0	17	5	BPP_RS11415-N142S and DNT-A1258V	56.5 % (13/23)	Clade 1
**1.2**	57	2	25	30	PtlD-V26M in 33 of 57 isolates	50.9 % (29/57)	Clade 2
**1.2.1**	152	0	24	128	PtlD-V26M and *prn*::delG-1895	98.7 % (150/152)	Clade 2

DNT: dermonecrotic toxin; PtlD: pertussis export protein D; *prn*: PRN gene. A SNP common to lineage 1 and sublineage 1.1 was the A425G nucleotide substitution within gene BPP_RS11415 (coding for a putative membrane monooxygenase), leading to a N142S change in the corresponding protein. This SNP was absent in isolates from sublineage 1.2 and clade 1.2.1 except for two isolates of sublineage 1.2 (BBP1_NCBI and B144) marked with a star on Fig. 1a.

Lineage 1 was defined in the broad sense as encompassing all isolates; but in the strict sense, ‘lineage 1’ will hereafter be used to design only the 18 isolates placed on early diverging branches of the phylogenetic tree. This lineage 1 contains more than 80% of the isolates collected before 1990 (Fig. 2b). All isolates of lineage 1, except the two most divergent ones, formed a deep phylogenetic branch characterized by two intergenic mutations (3,267,769 G>A and 3,267,889 C>T; described in more detail below) located between genes *bvgA* and *fhaB*. A monophyletic sublineage, called 1.1 (*n*=23 isolates), was defined by the mutation G3773A (leading to the amino acid change A1258V) within gene *dnt* encoding dermonecrotic toxin. This mutation occurred shortly before 1976 (95 % HPD: 1974, 1980); most isolates from this sublineage were collected between 1990 and 2009. Sublineage 1.2 (*n*=57) was defined as the sister group of 1.1, comprising the remaining isolates, and in its strict definition used hereafter, excluding clade 1.2.1. The monophyletic clade 1.2.1 (*n*=152 isolates) was defined by the mutation *prn*::delG-1895, which occurred shortly before this clade’s MRCA in 1998 (95 % HPD: 1996–2001) (Fig. 2a). Within sublineage 1.2, a mutation within gene *ptlD* (V26M change in the PtlD pertussis toxin export protein) was estimated to have occurred around 1987 (95 % HPD: 1984–1991) (Fig. 2a). It marked a single phylogenetic branch that included 33 isolates from sublineage 1.2 and the entire clade 1.2.1; these 183 isolates were collected between 1994 and 2018. For simplicity, we refer collectively to these four lineages/sublineages/clades as phylogenetic groups.

### SNP and insertion and deletion events

Of the 1994 SNPs, 265 were intergenic and 1729 were intragenic. Among the latter, 659 were synonymous and 1070 were non-synonymous (Table S2**)**. SNP densities in genes involved in regulation or coding for hypothetical proteins were statistically higher than the average (*p*<0.05), whereas SNP densities were statistically lower than average in genes involved in virulence or metabolism (*p*<0.05) (Supplementary Appendix, section Results: SNP densities per functional category paragraph; Table S3). A total of 69 SNPs were located within virulence-associated genes (details in Table S2 of the Supplementary Appendix).

We first analysed mutations related to virulence-associated genes. PT-related genes are present even though not expressed in *Bpp* because of the presence of several mutations in the promoter region [21]. We noted that all *Bpp* isolates displayed the same PT promoter *ptxP* sequence: all *Bpp* isolates present a *ptxP* promoter sequence 11 bp longer (CGCGGGATGCG) than the *Bp* one, with 23 mutations compared to the *Bp* allele *ptx*P1 (Fig. S6). In addition, SNPs were observed in several *ptl* genes encoding type IV secretion system (T4SS) components involved in PT secretion in *Bp* (including the V26M PtlD change; see Supplementary Appendix and Table S1).

We also observed 11 SNPs within the *fhaB* gene (locus tag BPP_RS15295), encoding FHA. Of these, six are non-synonymous, two of which affect the mature FHA [31]: one at position 3,261,750 (V1963A), observed in four isolates of lineage 1; and one at position 3,266,524 (T372A), observed in a single isolate (J324) of sublineage 1.2 (Table S2). nsSNPs were also observed to affect proteins functionally related to FHA, including FhaS, FhaJ and SphB1 (locus tag BPP_RS02120), a serine-protease involved in proteolysis maturation of FHA [41].

**Fig. 3.**
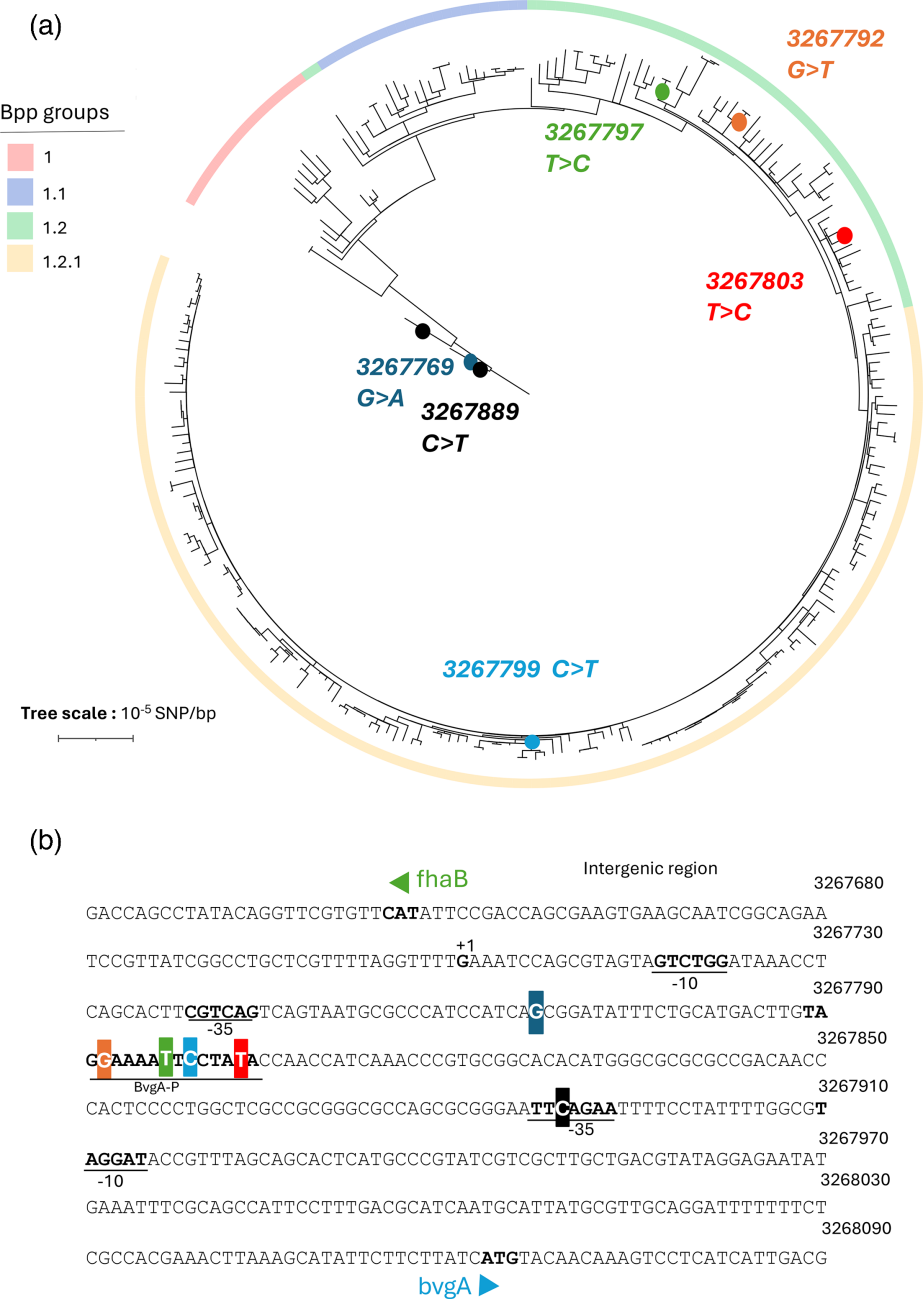
Mutations located in the intergenic region between *fhaB* and *bvgA*. Panel (a): Circular phylogenetic tree based on SNPs, rooted on isolate Bpp63.34. The four phylogenetic groups are represented with coloured branches as in Fig. 2. Lineage 1: light red; sublineage 1.1: light blue; sublineage 1.2: light green; clade 1.2.1: light yellow. Mutations observed within the *fhaB-bvgA* intergenic region are indicated by coloured circles on the tree branches and labelled with their nucleotide position in the genome. Panel (b): Localization of the observed mutations (highlighted with colour background as in panel a) within the intergenic sequence. The minus 10 and minus 35 motifs upstream of both genes, the +1 transcription start site of *fhaB* and the BvgA binding motif (from 3267789 to 3267804), are indicated in bold.

An exceptionally high SNP density was observed in the 425 bp intergenic region located between *fhaB* and *bvgA,* with six intergenic SNPs (Fig. 3; Table S3): (i) four are located in the phosphorylated BvgA binding site just upstream of the *fhaB* gene (also corresponding to P3 bvgA promoter) and were each observed in a few isolates collected after 2004; (ii) two others located at position 3,267,769 (G>A) and 3,267,889 (C>T) were largely shared by *Bpp* isolates. Indeed, both occurred in an early branch of the phylogeny (Fig. 2 and Fig. 3). Note that as the reference genome is within sublineage 1.2, these mutations are displayed as if they had occurred in the two early branching isolates, whereas in fact, in the early evolution of *Bpp,* it is the opposite mutations that occurred. These two mutations were fixed in extant *Bpp* populations, as isolates that do not carry these SNPs were not observed after 1958. While the first of these SNPs is located 23 nucleotides upstream of the −35 box of *fhaB*, the SNP at position 3,267,889 (C>T) is located within the −35 element of *bvg*A gene, changing the element from TT**C**AGAA to TT**T**AGAA, clearly suggesting an impact on gene expression (Fig. 3).

The PRN gene *prn* was also found to be highly impacted: only 51 of 250 isolates have a *prn* nucleotide sequence (locus tag BPP_RS05740) identical to the reference strain 12822; these included all isolates of lineage 1 (*n*=18 isolates/18), some isolates of sublineages 1.1 (*n*=8/23) and 1.2 (*n*=25/57, including the reference). For 192 of the 199 remaining isolates, the *prn* sequence had a point mutation, frameshift or insertion sequence mutation (Table S1**)**. We found a total of 18 distinct mutations. Four mutations were nonsense SNPs resulting in stop codons, whereas 13 were insertions or deletions events inferred to lead to PRN deficiency (Table S5). These distinct *prn*-disrupting mutations are scattered in sublineages 1.1, 1.2 and clade 1.2.1, and one of them (described above) is homoplasic (Fig. 2 and Supplementary Appendix, section Results: Homoplasic SNPs).

We next analysed the occurrence of insertions and deletions (INDELs). Regarding small INDELs, 329 INDEL events (as compared to the genome sequence of reference isolate 12822) were observed. Only 29 INDEL events were present in more than 10 isolates; 20 of these were located out of coding regions, and 9 within these (Table S4). Of these, seven were inferred to induce frameshifts, including two INDELs within the *prn* gene. Two INDELS were also observed within *fhaB,* but only affected three and one isolates, respectively (Table S4). As described above, the single G deletion in *prn* position 1895 (*prn*::ΔG-1895) was present in 150/152 from clade 1.2.1 (for H299 and H602 isolates, the analysis of the *prn* sequence did not allow us to confirm the presence of the mutation). This *prn*::ΔG-1895 deletion is by far the most frequently observed mutation within the *prn* gene, having been associated with the expansion of clade 1.2.1.

### Vaccine antigens production

PRN production status differed in the four lineages/sublineages/clades defined. All isolates from lineage 1 have a *prn* nucleotide sequence (locus tag BPP_RS05740) identical to the reference strain 12822, and so do 8 of 23 isolates of sublineage 1.1 and 25 of 57 isolates of sublineage 1.2 (including the reference). PRN production was confirmed experimentally for 31 of these 51 isolates (Table S1), being found positive as expected.

Among the 192 isolates with a point mutation, frameshift, or insertion or deletion events (Table S1**)**, the PRN deficiency was experimentally confirmed for the 125 isolates tested corresponding to all types of mutations (Table S1). Overall, 56.5 % (13/23) of sublineage 1.1 isolates, 50.9% (29/57) of sublineage 1.2 isolates and 98.7% (150/152) of clade 1.2.1 isolates were demonstrated or inferred to be PRN-deficient based on the presence of one of the 18 *prn* mutations.

FHA production was confirmed experimentally for 145 isolates using either Western blots or ELISA (Table S1). Using a selection of *Bpp* isolates with distinct mutations in the *fhaB-bvgA* region, the amount of FHA was evaluated using ELISA assays but did not reveal any difference according to mutational status (data not shown, available upon request; see Supplementary Appendix section Methods: Quantification of FHA expression in isolates with SNPs in the *fhaB-bvgA* intergenic region).

## Discussion

Acellular vaccines against *Bp* have been used for more than 20 years in multiple countries, including the USA, France and Spain. Here, we addressed the question of the possible impact that large-scale whooping cough vaccination might have exerted on the second agent of whooping cough, *Bpp*, even though this organism was not the explicit target of the vaccine. Because *Bpp* expresses two antigens, FHA and PRN, that are closely related orthologs of *Bp* vaccine antigens and are part of the *bvgAS* virulence regulon, changes in these proteins or their expression levels may have important implications on the transmission and pathogenesis of *Bpp* infections.

By taking advantage of a unique dataset of 250 human isolates collected over 83 years in three different countries, we also address the broader biological questions of the genomic diversity, population structure and genome-scale evolution of the so-far elusive *Bpp,* and the possible evolutionary parallelisms that this pathogen might show with *Bp*, its close relative and ecological competitor. Our data reveal, over time, the successive replacement of *Bpp* subpopulations by more recently emerged ones. By considering four deep *Bpp* phylogenetic groups, we showed how their relative proportions have shifted: whereas lineage 1 was predominant before 1990, clade 1.2.1 became the most frequent since 2010, now being nearly exclusive. This temporal replacement pattern is reminiscent of the disappearance of ancient lineages of *Bp,* which were replaced among extant infectious isolates by more recently evolved sublineages [12]. This scaled phylogenetic structure pattern is also typically encountered in human viruses evolving to escape previously built host immunity by antigenic drift, such as Influenza virus [42] or more recently SARS-CoV-2 [43]. The expansion of clade 1.2.1, characterized by a genomic rearrangement and the lack of PRN production, coincides with the peak detected in the effective population size analysis, around the year 2010, suggesting that isolates from clade 1.2.1 may have a better fitness in the three surveyed countries using acellular vaccines, as observed for *Bp* PRN-negative isolates [18]. The evolution of *Bpp* by successive phylogenetic group replacement might be driven by a combination of its ongoing adaptation to humans, natural immunity built in human populations as a result of infection, ecological interactions with *Bp* [44,45] and possibly also by vaccination-induced immunity, which we discuss below. Defining whether the mutational changes that mark defined phylogenetic groups caused a change in *Bpp* biology would require experimental investigations.

The *Bpp* population genomics data also revealed a regular pattern of SNP accumulation over time, enabling us to estimate the mutation rate of *Bpp* at 2.1×10^−7^ substitutions per site·year^−1^. This rate is remarkably similar to the one estimated within the main branch of *Bp* (2.24×10^−7^ substitutions per site·year^−1^) [12], consistent with the shared recent ancestry and similar ecology of these two pathogens, which both evolved from their progenitor genomic species *B. bronchiseptica* [12,25, 39]. Remarkably, the estimated MRCA of human *Bpp*, around the year 1877, is more recent by only a few decades than the main *Bp* branch, which was estimated to have emerged between the years 1790 and 1810 [12]. The crowding and promiscuity that increased rapidly during the industrial revolution in the nineteenth century represent a possible driver of the expansions of *Bpp* and *Bp* in the populations of developed countries. Clearly, our sample (mainly from three Northern hemisphere countries) may miss deeper branching isolates that could be circulating elsewhere or have become extremely rare, similar to the exceptionally rare deep lineage of *Bp* [12]. Note that ovine *Bpp* isolates, which were seldom reported and for which only a single genomic sequence is available [10,46, 47], were not considered in this study because they belong to a separate evolutionary lineages [25] and, hence, would not affect the above temporal analyses and conclusions.

*Bpp* does not produce PT (produced in *Bp* from the PT gene cluster, *a.k.a.* the *ptx-ptl* operon), which has been attributed to mutations observed within a few *Bpp* strains in the promoter sequences of the synthesis gene cluster [21,48]. Here, we confirm the presence of these mutations at population scale, and several additional mutations were uncovered in the coding sequences of the *ptx-ptl* gene cluster. However, although several nsSNP were found, there was no compelling evidence for gene decay. Similarly, fimbriae FIM2 and FIM3 proteins are not expressed in *Bpp*, but their genes are present and undisrupted in *Bpp* genomes [22,40]. Here, no SNP was observed within fimbriae genes: neither within *fimABCD* structural genes nor within *fimX* or *fimN* genes, which code for other fimbriae subunits. As for the PT gene cluster, a lack of obvious gene decay at population level may suggest the intriguing possibility of a cryptic role for these clusters, which are perhaps expressed in yet undefined conditions in *Bpp* [22].

Regarding the effect of whooping cough vaccination on *Bpp*, our work uncovers genetic signatures of evolution in the genes coding for the two *Bp* vaccine antigens PRN and FHA which are expressed by *Bpp*. FHA is one of the components of most (but not all) current aPVs, and BvgA is the main regulator of virulence genes in *B. bronchiseptica* and *Bp*. The *bvgA-fhaB* intergenic region was previously shown to have undergone extensive evolution in *Bp* [12], which may have impacted not only the expression of both genes, but also those of the *bvgAS* regulon [49]. Here, we report six mutations located in the orthologous intergenic region of *Bpp*. One of these mutations (in position 3,267,792 in Fig. 3 or 155 in Fig. S3) is at the exact same position as a mutation observed in *Bp* [12,40]. Two SNPs in the intergenic *bvgA-fhaB* region occurred early (Fig. 2; estimated around 1909) in the evolutionary history of *Bpp* and became fixed in extant populations. These two mutations predate largely pertussis vaccination and may have been selected for adaptation to humans, who were recent novel hosts for *Bpp* at that time, or in reaction to natural infection-driven immunity. The high SNP density in the *bvgA-fhaB* intergenic region in both *Bp* and *Bpp* suggests a central role of this critical regulatory region in evolutionary adaptation to the human niche, as both pathogens diverged from their ecological generalist progenitor species *B. bronchiseptica*. Whether and how the two ancestral *Bpp* intergenic mutations have impacted the levels of *fhaB* expression, *bvgA* expression, or both, thus stands out as a central question to decipher the adaptive trajectory of *Bpp*, which should be addressed experimentally in future studies.

We confirmed FHA production experimentally in all tested *Bpp* and failed to show a link between mutations in the *fhaB-bvgA* intergenic region and expression levels (see Supplementary Appendix, Methods section). Additional non-synonymous genetic variation in *fhaB* and functionally related genes was also observed (Supplementary Appendix, Results section). As FHA-negative *Bp* are exceptional too [28], this protein seems to exert an essential role in the biology of both agents of whooping cough. Overall, these observations point to a particular role of FHA and related functions in *Bpp* biology, as in *Bp* [50].

In *Bp*, PRN deficiency is a major recent evolutionary phenomenon, shown to be driven mainly by acellular vaccine (aPV)-induced immunity [14,41, 51]. But so far, the genetic evolution of PRN expression in *Bpp* has been little documented [52,53]. Our data provide strong evidence for the evolution of this antigen being driven by aPV too. First, we observed a population shift towards PRN deficiency in *Bpp*, which started just after the rollout of aPVs. Second, besides the prominent *prn*::ΔG-1895 mutation, 17 other PRN deficiency mutations were identified, and all were dated between 2005 and 2018. This convergent pattern of gene disruption after the introduction of aPV strongly supports the view that PRN expression by *Bpp* is disadvantageous in aPV countries. Data from three countries that use wPV instead of aPV further support this hypothesis, as no PRN deficiency was observed in *Bpp* isolates collected from these countries between 1998 and 2015 [31,54, 55]. Although more *Bpp* sampling would strengthen the trend we observed, the PRN-deficient population increase seems to be even faster in *Bpp* than observed for *Bp* (Fig. S4), as almost all *Bpp,* but only 50–90% *Bp*, depending on country, are now PRN deficient [56,57], suggesting a strong selective advantage of PRN-deficient *Bpp*.

Overall, our genomic analyses indicate that, even though pertussis vaccines were designed against *Bp*, *Bpp* has indeed been affected by pertussis vaccination. Although the phylogenetic proximity and shared antigens of *Bp* and *Bpp* make the ‘bystander’ status of *Bpp* questionable, our work uncovers a clear evolutionary impact of vaccination on an organism that was not the explicit target of the vaccine. In the strict sense, this work thus demonstrates a bystander impact of vaccination on a non-target organism.

Vaccination against *Bp* has been considered to have low, or even no, efficacy against *Bpp* [23,58, 59]. However, the extent of PRN deficiency that we detected amongst *Bpp* isolates in aPV vaccinated populations suggests that the vaccines may provide some cross-protection, as PRN production seemingly incurs some fitness cost [14]. An important implication is that, as extant isolates of *Bpp* now rarely produce PRN, cross-protection against *Bpp* from whooping cough vaccines may have weakened significantly in the last 20 years. This evolution leaves only FHA as an aPV vaccine antigen expressed by *Bpp*, against which the bactericidal activity of antibodies is weak [51]. Future improved whooping cough vaccines could benefit from comprising *Bp-Bpp* cross-reacting antigens explicitly, such as the adenylate cyclase [60] or conserved antigens identified through immuno-informatics [61], or could incorporate *Bpp*-specific antigens, such as the O-antigen [62].

In conclusion, our study provides important novel insights into the past evolutionary dynamics of *Bpp* and uncovers a remarkable picture of parallel evolution between the adaptation of *Bpp* and *Bp* populations to humans, including their timing of emergence, rate of evolution, successive lineage replacement, early adaptation to the human niche and vaccine-driven evolution. These parallelisms illuminate how two distinct pathogens that have evolved from a single common ancestral species have adapted to the human host and later in response to vaccination-induced immunity. The deep evolutionary picture we uncovered for *Bpp* highlights the bystander effect of pertussis vaccination against *Bpp* as the latest example of the evolutionary parallelism between the two agents of whooping cough.

## Supplementary material

10.1099/mgen.0.001544 Uncited Supplementary Material 1.

10.1099/mgen.0.001544 Uncited Table S1.

10.1099/mgen.0.001544 Uncited Table S2.

10.1099/mgen.0.001544 Uncited Table S3.

10.1099/mgen.0.001544 Uncited Table S4.

10.1099/mgen.0.001544 Uncited Table S5.

## Abbreviations

aPVs: acellular pertussis vaccines
*Bp*: *Bordetella pertussis*
*Bpp*: *Bordetella parapertussis*
CDC: Centers for Disease Control and Prevention
FHA: filamentous hemagglutinin
FIM2: type 2 fimbriae
FIM3: type 3 fimbriae
HPD: highest posterior density
INDELs: insertions and deletions
MRCA: most recent common ancestor
nsSNP: non-synonymous mutations
PFGE: pulsed-field gel electrophoresis
PRN: pertactin
PT: pertussis toxin
SNP: single nucleotide polymorphism
wPVs: whole-cell pertussis vaccines
