# Supplementary material for: No innocent bystanders: pertussis vaccination and evolutionary parallelisms between Bordetella parapertussis and Bordetella pertussis
Source: Microb Genom. 2025 Nov 14;11(11):001544. doi: 10.1099/mgen.0.001544 (PMC12617973; doi:10.1099/mgen.0.001544)
Supplement: Uncited Supplementary Material 1. [file mgen-11-01544-s001.pdf]

Supplementary Appendix

No innocent bystanders: pertussis vaccination and evolutionary parallelisms between *Bordetella parapertussis* and *Bordetella pertussis*

Valérie Bouchez[a,b], Albert Moreno-Mingorance[c], Alba Mir-Cros[d], Annie Landier[a,b], Nathalie Armatys[a,b], Sophie Guillot[a,b], Maria Teresa Martín-Gómez[e], Carla Rodrigues[a,b], Julie Toubiana[a,b,f], Ana I. Bento[g], Michael R. Weigand[h], Juan José González-López[c,d,e,i]# and Sylvain Brisse[a,b]#

[a] Institut Pasteur, Université Paris Cité, Biodiversity and Epidemiology of Bacterial Pathogens, Paris, France.

[b] Institut Pasteur, National Reference Center for Whooping Cough and Other Bordetella Infections, Paris.

[c] Vall d'Hebron Institut de Recerca (VHIR), Vall d'Hebron Hospital Universitari, Vall d'Hebron Barcelona Hospital Campus, Barcelona, Spain

[d] CIBER de Enfermedades Infecciosas (CIBERINFEC), Instituto de Salud Carlos III, Madrid, Spain.

[e] Department of Clinical Microbiology, Hospital Universitari Vall d'Hebron, Barcelona, Spain.

[f] Université Paris Cité, Department of General Pediatrics and Pediatric Infectious Diseases, Hôpital Necker–Enfants Malades, APHP, Paris, France.

[g] Department of Public & Ecosystem Health, College of Veterinary Medicine, Cornell Univ, Ithaca, NY, USA.

[h] Division of Bacterial Diseases, Centers for Disease Control and Prevention, Atlanta, Georgia, USA.

[i] Department of Genetics and Microbiology, Universitat Autònoma de Barcelona, Barcelona, Spain.

Contents:

Supplementary Methods **Page 2**
Supplementary Results **Page 4**
Supplementary Tables list **Page 10**
Supplementary Figures **Page 11**
Supplementary References **Page 16**

Supplementary Methods

Microbiological characterization

Isolates were grown at 36 °C for 72 h on Bordet Gengou Agar (BGA, Becton Dickinson) supplemented with 15% defibrinated sheep blood (ThermoFisher Diagnostics) and stored in BSA/saccharose–phosphate–glutamate (BSA/SPG) solution at -80 °C until further use. Isolates were characterized using classical bacteriological methods, including observation of hemolysis as well as oxidase and urease tests. For identification, API20E biochemical strips were used until 2014 and MALDI-TOF mass spectrometry was used since 2015.

For antigen characterization of French isolates by Western blotting, pulsed-field gel electrophoresis (PFGE) and DNA preparation, isolates were sub-cultured for 24 h in BGA medium. Bacteria were then resuspended in physiological salt to reach an optical density at 650 nm of 1 (called the OD1 suspension). Pertactin and filamentous hemagglutinin (FHA) production of French isolates was evaluated from bacteria contained in 0.6 ml of OD1 suspension by the Western blot method using corresponding antibodies prepared with *Bp* antigens (in house preparation after mouse immunization with purified antigens) [1]. PFGE restriction profiles were obtained using the *Xba*I enzyme [1].

For the Spanish isolates, pertactin and FHA production was evaluated by indirect whole-cell enzyme-linked immunosorbent assay (ELISA), using two specific monoclonal anti-pertactin (97/558, NIBSC) and anti-FHA (99/572, NIBSC) antibodies [2].

Quantification of FHA expression in isolates with SNPs in the *fhaB-bvgA* intergenic region

To quantify FHA production in isolates with various states of mutations in the *fhaB-bvgA* intergenic region, we included 18 isolates, comprising those with intergenic SNPs identified between *fhaB* and *bvgA*, as well as genetically closely related isolates as phylogenetic controls. Production of FHA was evaluated by indirect whole-cell enzyme-linked immunosorbent assay (ELISA) using a monoclonal antibody (mAb) specific to FHA of *B. pertussis* (99/572; National Institute for Biological Standards and Control, https://www.nibsc.org), as previously described by Barkoff *et al.* [2]. In the assay, one French FHA-negative *B. pertussis* isolate (FR4624) was used as negative control, and one Spanish FHA-positive *B. pertussis* isolate (HJXXIII-057) was used as a positive control.

DNA preparation and genomic sequencing

For isolates from France, 400 µL of each OD1 suspension were pelleted and suspended in 100 µL of 1X phosphate-buffered saline, 100 µL of lysis buffer (Roche Diagnostics, Meylan, France), and 40 µL of proteinase K; heated at 65°C for 10 min and then at 95°C for 10 min. Libraries were constructed using the Nextera XT DNA Library Preparation kit (Illumina, San Diego, CA). Sequencing was performed on a NextSeq-500 sequencer (Illumina, San Diego, CA) using a 2×150 paired-end protocol at the Institut Pasteur Mutualized Platform for Microbiology (P2M) as previously described [3].

For isolates from Spain, DNA extraction was performed with DNeasy UltraClean Microbial Kit (Qiagen, Spain). For whole genome sequencing, DNA libraries were constructed using a Nextera DNA Flex Library Preparation kit (Illumina, San Diego, CA) and sequenced using a MiSeq System (Illumina, San Diego, CA), through a 2x300 paired-end protocol.

For isolates from the USA, genomic DNA was prepared with the Gentra Puregene yeast/bacteria kit (Qiagen, Valencia, CA) as previously reported [4]. Whole-genome shotgun sequencing of isolates was performed using a combination of the PacBio RSII (Pacific Biosciences, Menlo Park, CA), Illumina HiSeq/MiSeq (Illumina, San Diego, CA), and Argus (OpGen, Gaithersburg, MA) platforms as described previously [5].

Effective population size dynamics

The software tool BEAST version 1.10.4 [6] was used to infer the effective population size dynamics. HKY, strict clock and Bayesian Skygrid were used to infer temporal and demographic parameters (details in Supplementary material). The Skygrowth R package [7] was used to infer and compare the effective population size predicted by BEAST. Ten percent randomly selected samples were deleted from the tree, three times, to infer the sampling robustness of the analysis. Tree annotation with relevant metadata was performed with ggtree and ggplot R packages [8].

Model selection was achieved by comparing the marginal likelihoods estimated using path sampling. Various substitution models, clock models and coalescent priors were compared with 200 million chains. The strict clock and the uncorrelated log normal clock were tested, and coalescent constant population, Bayesian Skyline, Bayesian Skyride and Bayesian Skygrid tree priors were studied. As result, HKY, strict clock and Bayesian Skygrid were used to infer temporal and demographic parameters.

Supplementary Results

Characteristics of four main *Bpp* phylogenetic groups

We defined four successive *Bpp* phylogenetic groups within the phylogenetic tree (see main text).

Lineage 1 was defined in the broad sense as the global lineage englobing all isolates; but to distinguish its early-diverging branch from its nested groups defined hereafter, in the strict sense, lineage 1 was used to design only the 18 isolates placed on early diverging branches of the phylogenetic tree. They were collected mainly between 1963 and 1993, and all possess an intact *prn* gene. All but two early-diverging isolates have the two ancestral mutations in the *bvgA-fhaB* intergenic region.

Sublineage 1.1 corresponds to all isolates (n=23) with the mutation G3773A, leading to amino acid change A1258V, within gene *dnt* (locus tag BPP_RS17875). These isolates were collected between 1993 and 2016. Of 21 isolates for which pertactin status could be determined, 8 isolates have an intact *prn* gene, whereas 13 have a mutation within *prn* (locus tag BPP_RS05740): 4 with *prn*::del-4bp-2208; 7 with *prn*::delA-988; 1 with *prn*::del-5bp-75 and 1 with *prn*::Stop-C2533T. For the 2 remaining isolates, it was not possible to determine the mutation within *prn* gene from the genomic sequence.

A common SNP to lineage 1 and sublineage 1.1 was the A425G nucleotide substitution within gene BPP_RS11415, leading to the N142S change. This change (compared to the reference genome, which belongs to lineage 1.2) was absent in isolates from lineage 1.2 and clade 1.2.1 except for 2 isolates of lineage1.2 (BBP1_NCBI and B144, marked by a back star on Figure 1). Because the reference genome belongs to lineage 1.2, the corresponding evolutionary change can be deduced to be S142N, which occurred just after the divergence of the two early lineage 1.2 isolates BBP1_NCBI and B144.

Lineage 1.2 (n=55) contained (in the strict sense) all non-lineage 1 and 1.1 isolates, which did not have the *prn*::delG-1895 mutation. Whereas 25 isolates of lineage 1.2 have an intact *prn* gene, 27 display *prn* mutations (10 with *prn*::delG-784, 4 with *prn*::delG-283, 5 with insertions, 6 with Stop codons, and 2 with *prn*::C<T-1168); note that the *prn* gene sequence of the three remaining isolates was undetermined.

Clade 1.2.1 (n=152) was defined within sublineage 1.2 (in the broad sense) as comprising isolates with the pertactin expression disruption mutation *prn*::delG-1895 (the *prn* gene sequence of 2 isolates was undetermined). All 1.2.1 isolates were collected after 2005.

Effective population size analysis

Based on our dataset, we performed effective population size (EPS) analysis. As differences in sampling (between countries or over time) can bias demographic reconstructions, we first performed country-specific effective population analysis. **Fig. S2A** included isolates collected in France through the RENACOQ network, for which the sampling design has been stable since 1996. It showed a temporary decrease of the EPS around 2005, corresponding to the switch to acellular vaccines in France, and then a population size recovery and then further increase concomitant with the appearance of pertactin-negative strains (see **Fig. S4**). This suggests the interesting possibility that this demographic analysis captures the strong selective advantage effect of pertactin loss on *Bpp* fitness in human populations vaccinated with acellular vaccines containing pertactin. Although it would be interesting to explore this effect based on the other datasets, the lack of systematic sampling from the two other countries prevents us for drawing strong conclusions.

When analyzing isolates collected in the USA by the CDC (**Fig. S2B**), we observed an overall increase in effective population size until 2008, when it started to decrease. However, by that time, pertactin-negative isolates were already predominant in this country. As in France, a similar overall increasing long run trend was observed in the USA. Data from Spain were too scarce and not systematically sampled either.

We analyzed the overall population, comprising isolates from the three countries and public genomes (**Fig. S2C)**. We performed sensitivity analyses, where we restricted the analysis to subsets of the data applying repeated 90% random subsampling (**Fig. S2D**). The inferred demographic patterns suggested an increase from year 1950 (2020-70) till year 1970 (2020-50), then decreased until 1995 (2020-25), then increased again until 2008. The relative contributions to this pattern of true population size variation or sampling heterogeneity over time, is difficult to ascertain.

Importantly, the uncertainty around the reconstructions is explicitly represented in the credibility intervals shown in the figures, which naturally widen in periods or regions with lower sampling density indicating that the signal is strong. Further the effective sample sizes (ESS) from our runs are high (>200 in Tracer), which supports reliable inference under a simple model. In summary, the combination of (i) multiple inference frameworks (Skygrid and Skygrowth), (ii) robustness analyses by geography and subsampling, (iii) explicit reporting of uncertainty, and (iv) the broad temporal and geographic representation of our dataset, together provide a balanced demographic inference. Longitudinal data from other settings will be needed to provide more insights into *Bpp* demographics in the future.

SNP densities per functional category and Bvg label

We classified coding sequences (CDS) having homologs in *Bp* as in Bart et al. [9], into 20 functional categories completed by an additional category comprising LPS-structural genes. Indeed, contrary to *Bp*, which has a cell wall lipooligosaccharide (LOS), *Bpp* displays a whole lipopolysaccharide (LPS) structure. Intragenic SNPs were labelled according to these categories (**Table S2**). We also labelled genes into "Bvg-activated" and "Bvg-repressed" categories, based on published data [10,11]. SNP densities in each category were calculated as the ratio between polymorphic sites and the sum of involved CDSs lengths (**Fig. S5, Table S3)**. Fisher's exact test was used to test for differences in SNP densities.

Compared to the SNP density of all CDSs with a functional classification (1.04 ‰ SNPs/bp on average), the SNP densities within the "virulence-associated genes" category (0.72 ‰ SNPs/bp, p=0.0023) and the "metabolism" category (0.91 ‰ SNPs/bp, p=0.04) were lower. In contrast, SNP density was higher in the "hypothetical protein" and "regulation" categories (1.3 ‰ SNPs/bp, p=0.04 and 1.3 ‰ SNPs/bp, p=0.03). The two categories related to *bvg* regulation had a SNP density close to the average (respectively 0.94 ‰ for *bvg*-activated and 1.08 ‰ SNPs/bp *bvg*-repressed genes; p>0.05 in both cases).

Homoplasic SNPs

Only 2 of the 1,994 SNPs were homoplasic. The first was located at position 1,231,601 within the *prn* gene (locus tag BPP_RS05740), corresponding to a nonsense mutation leading to a stop codon (Q845*Stop) **Table S1&S2**). This SNP was found in 2 isolates (H102 collected in the USA in 2009; and BPP305 collected in Spain in 2007) belonging to groups 1.1 and 1.2, respectively. The occurrence of this *prn*-disrupting SNP in independent branches is consistent with a selective advantage of losing pertactin expression.

The second homoplasic SNP was at position 2,966,333 in the intergenic region between gene BPP_RS13990, coding for a GntR family transcriptional regulator, and gene BPP_RS13995, coding for a hydrolase. This SNP was found in 4 isolates, all located in sublineage 1.2; and occurred in two branches: one containing H904 collected in the USA in 2012; and the other containing BPPG20 (2018, Spain), E765 (2004, USA) and I457 (2012, USA).

Variation in genes associated with lipopolysaccharide, toxins and autotransporters

Lipopolysaccharide (LPS) is present at the surface of *Bpp*, contrary to the situation in *Bp*, which has no LPS. Thus, the variation in the genes that direct its synthesis might reveal unique adaptive clues of *Bpp*. 18 SNPs were located in genes related to LPS-structural genes. We observed 11 SNPs located in the *wbm* locus responsible for the synthesis and expression of the O antigen. Five of them, in locus tags BPP_RS00655, BPP_RS00690, BPP_RS00730 and BPP_RS00740, were observed in a few isolates of sublineages 1.1, 1.2 or of clade 1.2.1, and were non-synonymous (**Table S2**). In addition, 6 non-synonymous SNPs (nsSNPs) were located in the *wlb* locus (previously called *bpl* locus) coding for the biosynthesis of the LPS trisaccharide in locus tags BPP_RS00745, BPP_RS00750, BPP_RS00760, BPP_RS00770 and BPP_RS00785 (**Table S2**). We also report mutations in genes involved in LPS modification such as *lpxA* (locus tag BPP_RS07700); *lpxC* (locus tag BPP_RS18890) and in LPS export *lptC* gene (locus tag BPP_RS20345) but only in few isolates.

Regarding the pertussis toxin-related genes, which are present even though not shown to be expressed in *Bpp,* we noted that all *Bpp* isolates displayed the same pertussis toxin promoter *ptxP* sequence [12]: all *Bpp* isolates (as the 12822 reference strain) present a *ptxP* promoter sequence 10 bp longer (CGCGGGATGCG) than the *Bp* one, and have 23 point mutations compared to Bp *ptx*P1 sequence (**Fig. S6**). *Bp* isolates with a *ptxP3* type promoter have been suggested to produce more PT than *Bp* isolates with a *ptxP1* type promoter sequence [13]. Only one mutation distinguishes *ptxP1* and *ptxP3* promoters in *Bp*, and in *Bpp* the *ptxP* promoter at this position has the same base as *ptxP1.*

In addition, SNPs were observed in several *ptl* genes encoding type IV secretion system (T4SS) components involved in PT secretion in *Bp*: first, a SNP leading to a V26M change in PtlD (locus tag BPP_RS21745) was observed in all isolates of sublineage 1.2 and clade 1.2.1. Further, six additional mutations, which were restricted to few isolates, were observed within others *ptl* genes, of which four were non-synonymous: in *ptlB* (locus tag BPP_RS21735, D59N, in sublineage 1.2 isolate Bpp305), *ptlD* (T127M in lineage 1 isolate Bpp006, and G197A in lineage 1 isolate FR3638), and in *ptlH* (locus tag BPP_RS21770, A62T) in lineage 1 isolate Bpp0031).

Regarding the dermonecrotic factor DNT, in addition to the sublineage 1.1 landmark *dnt* SNP leading to the A1258V change described above, other nsSNPs were observed: P1132L in clade 1.2.1 isolate J217 and H1074Y in 8 isolates of clade 1.2.1. The dermonecrotic factor (DNT) is a virulence factor regulated by *bvg*AS and produced by the three classical *Bordetella* species [14], except for ovine *Bpp* [15]. DNT is an important virulence factor of *Bbs*, involved

in pig atrophic rhinitis [16] and may contribute to encephalopathy by *Bp* [17]. Nothing is known about DNT function in *Bp* or *Bpp*, but the two nsSNPs found here may reflect adaptation of *Bpp* sublineage 1.1 to humans.

In contrast, adenylate cyclase toxin, which is encoded by the *cyaA* gene (locus tag BPP_RS01640) was highly conserved in *Bpp.* We found only 3 SNPs that affected 5 isolates. Two were nsSNPS: I217N in BPP012 isolates from sublineage 1.2 and S1173T in 3 isolates from clade 1.2.1 (H602, J778 and J835).

Autotransporters and the type 3 secretion system (T3SS) play an important role in *Bordetella* virulence. In addition to the SNPs already mentioned above within *prn*, additional nsSNPs were observed in *Bpp* genes coding for *sphB1* (see below), *batB* and *bscR* and T3SS genes (**Table S2**).

FHA variation in FHA and functionally related genes

Besides the two ancestral *bvgA-fhaB* intergenic mutations, the four other *bvgA-fhaB* intergenic SNPs are located in the phosphorylated BvgA binding site of the *fhaB* promoter [9,18], suggesting a functional impact of these mutations. However, these were observed in only a few isolates, and may thus reflect a transient selective advantage, perhaps in patients with atypical anti-FHA immunity.

Mutations in the coding sequence of *fhaB* may reflect the fine-tuning of FHA protein interaction with its receptor, even though none were located in the FHA-RGD motif. Several mutations also occurred in *Bp* within the gene encoding FHA [9,19].

SphB1 (locus tag BPP_RS02120), a serine-protease involved in proteolysis maturation of FHA [20], had a nsSNP at position R185L in an isolate from clade 1.2.1 (FR6071). We also observed nsSNPs in two other genes encoding FHA-like proteins, *fhaS* (locus BPP_RS06200) and *fhaL* (locus BPP_RS12565) [21,22]: *fhaS* F979S in one isolate of clade 1.2.1 and *fhaL* (P2845L, A2508E and T2008I), each in one isolate, belonging to sublineages 1.2 and 1.2.1. Most of these mutations are restricted to a small number of *Bpp* isolates.

Genomic structure and rearrangements

Genomic rearrangements had previously been reported for *Bpp,* all bordered by IS*1001* or IS*1002* [23,24]. PFGE profile variations were previously reported in *Bpp* [5,25]. Here, PFGE restriction profiles were obtained using the *Xba*I enzyme [6] for 84 isolates from France and the reference strain 12822. The data revealed the existence of two PFGE profiles labeled I and II (**Fig. S7**), consistent with previous results [6]. PFGE-I was found in 33/85 isolates, all from

sublineages 1.1 and 1.2, including pertactin-producing and pertactin-deficient isolates. PFGE-II was observed in 52 of 85 isolates from clade 1.2.1.

Genomic structure data was available for 74 US isolates and for reference strain 12822, for which the genome assembly was circularized [5]. Two main clusters, PP-01 and PP-02, were previously reported, with a re-arrangement between them corresponding to a symmetric inversion between copies of IS*1001* from position 1,316,955 to 3,351,187 [5].

The phylogenetic distribution of rearrangements was congruent with PFGE profiles: most PFGE-I isolates corresponded to cluster PP-02, whereas most PFGE-II isolates were in the same branch as cluster PP-01 isolates (**Fig. S7**). These results show that a single major genomic rearrangement, resulting in a symmetric inversion between copies of IS*1001* [24], occurred in the evolution of *Bpp*, and that this rearrangement was previously captured by PFGE analysis. This rearrangement became nearly fixed in extant *Bpp* isolates, including those from clade 1.2.1.

Insertion sequences

We found that the copy number of insertion sequences in *Bpp* genomes is stable: We found 22 copies of IS*1001* and 9 copies of IS*1002* in respectively 225 and 231 assemblies. Only few assemblies had different copy numbers of IS*1001* and IS*1002* (**Table S1**). This is an important observation, as *Bpp* diagnosis is performed using qPCR targeting IS*1001* defined from the reference genome [23]. Our results mean that detection of *Bpp* should not be affected by the observed genomic variation.

Supplementary Tables

Table S1: Characteristics of isolates.

Table S2: Single nucleotide polymorphisms (SNPs) identified in *B. parapertussis.*

Table S3: SNP densities according to functional categories and *bvg* status.

Table S4: Insertions and deletions detected by Snippy in all samples against BPP12822.

Table S5: Pertactin sequence variants observed in *Bordetella parapertussis* isolates.

Supplementary Figures

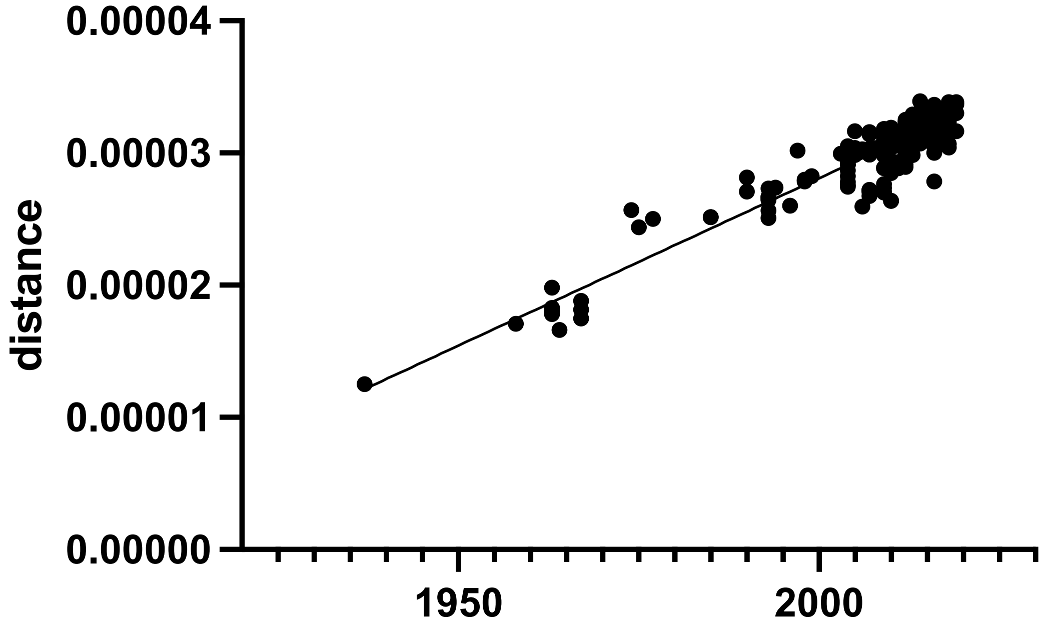

Figure S1. Root-to-tip genetic divergence *versus* time. The graph was created using TempEst based on the 250 *B. parapertussis* isolates collected in the period 1937-2019.

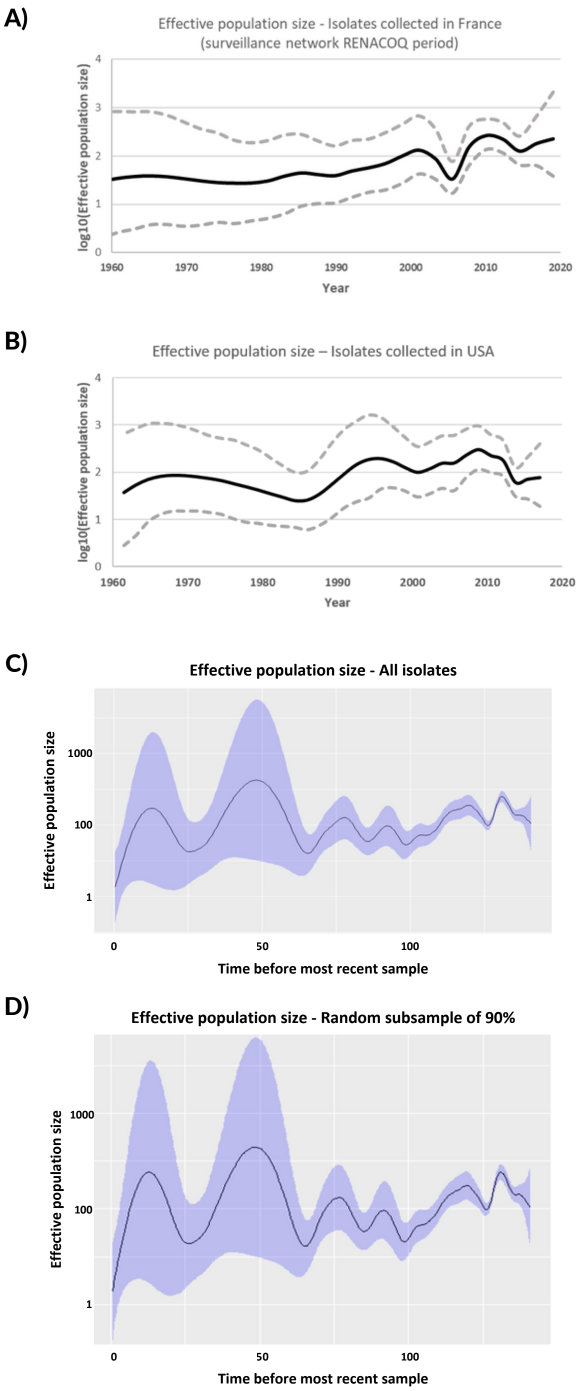

Figure S2. Effective population size analysis. **A.** Effective population size of *B. parapertussis* inferred in the Bayesian phylogenetic reconstruction conducted using strict clock and Bayesian Skygrid model analyzing isolates collected in France. **B.** Same, for isolates collected in the USA. **C**. Robustness analysis of effective population size of *B. parapertussis* inferred with Skygrowth R package. **D.** Robustness analysis of effective population size of *B. parapertussis* inferred with Skygrowth R package, performed with random subsamples of 90% of isolates.

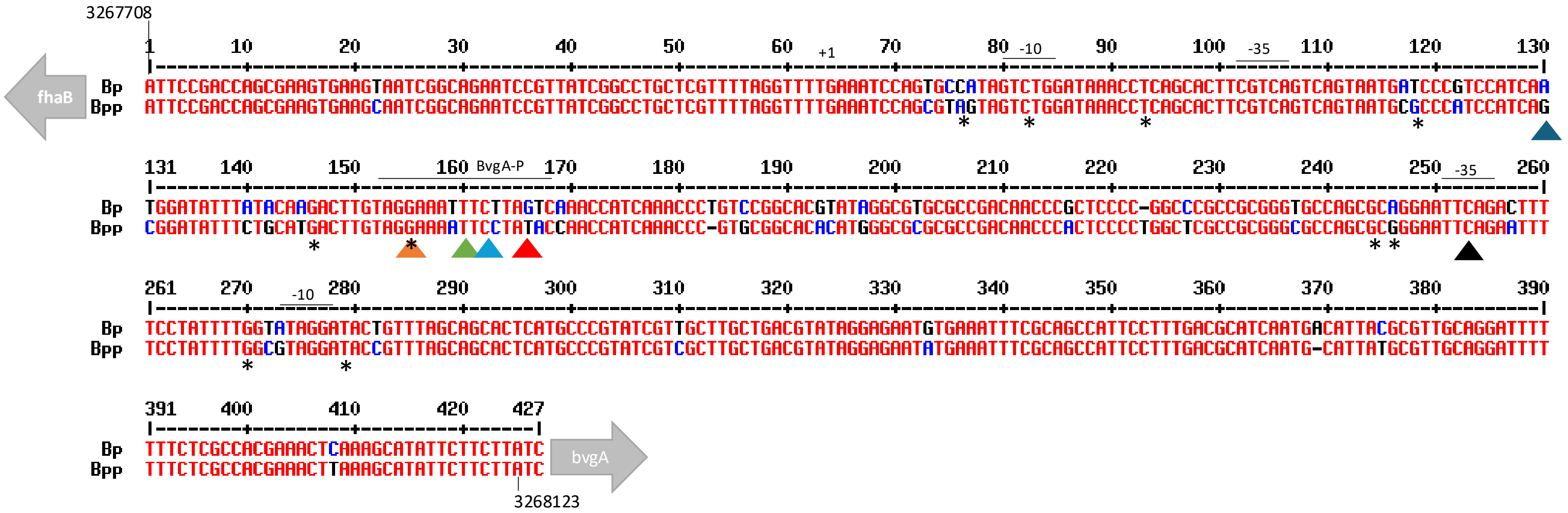

Figure S3. Mutations within the *bvgA-fhaB* intergenic sequences. *B. pertussis* (Black stars below the lines indicate mutations found in *Bp* isolates *vs* strain Tohama) and *B. parapertussis* (Colored triangles for mutations found in Bpp isolates *vs* strain 12822) sequences are shown aligned. Triangle colors for mutations found in *Bpp* are the same as in **Fig. 3** (*dark blue 3267769 G>A; orange 3267792 G>T; green 3267797 T>C; light blue 3267799 C>T; red 3267803 T>C and black 3267889 C>T). Positions 3267708 and 3268123 refer to Bpp genome as in Figure 3.*

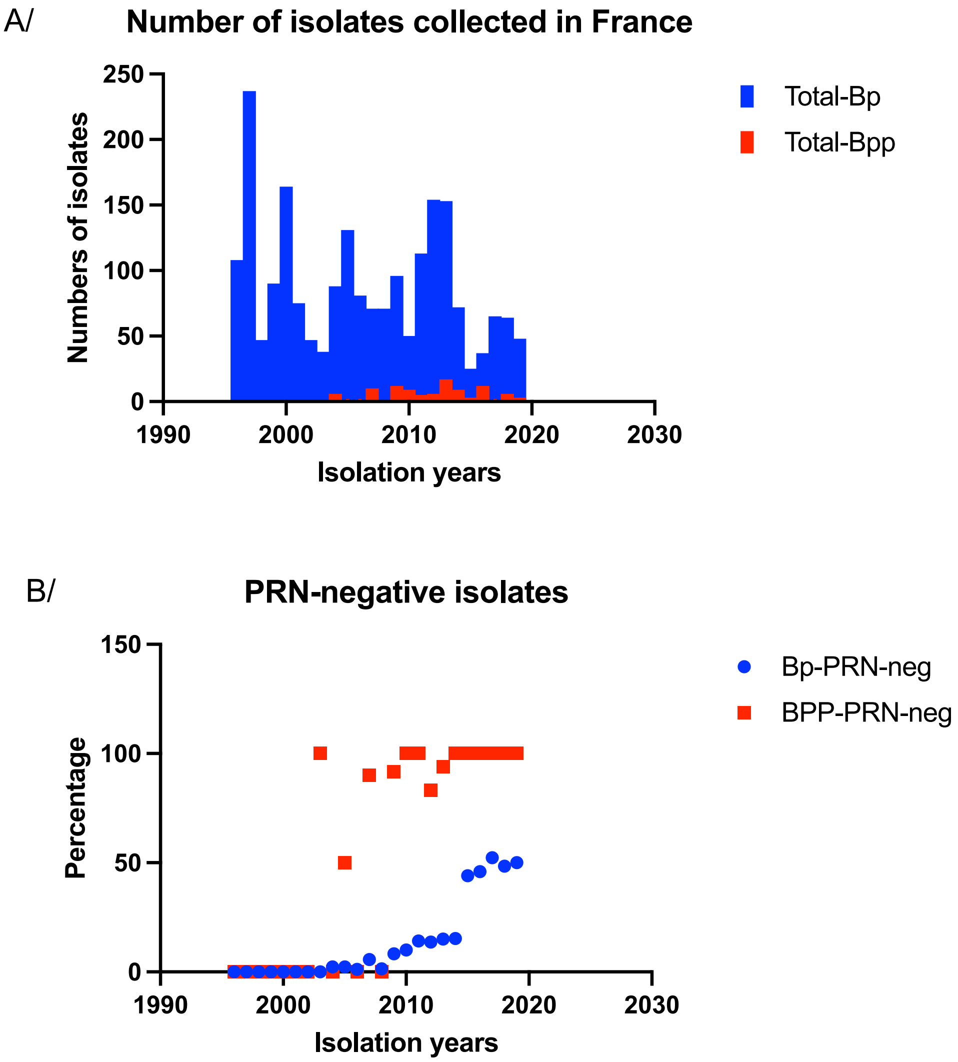

Figure S4. *Bordetella pertussis* (Bp) and *B. parapertussis* (Bpp) isolates collected in France, per year (1996-2019).

Panel A: Numbers of collected isolates, per year. Bp isolates: blue bars; Bpp isolates: red bars. Panel B: Percentage of pertactin-negative isolates. Pertactin-negative Bp: blue circles, pertactin-negative Bpp: red squares. PRN-neg: pertactin-negative.

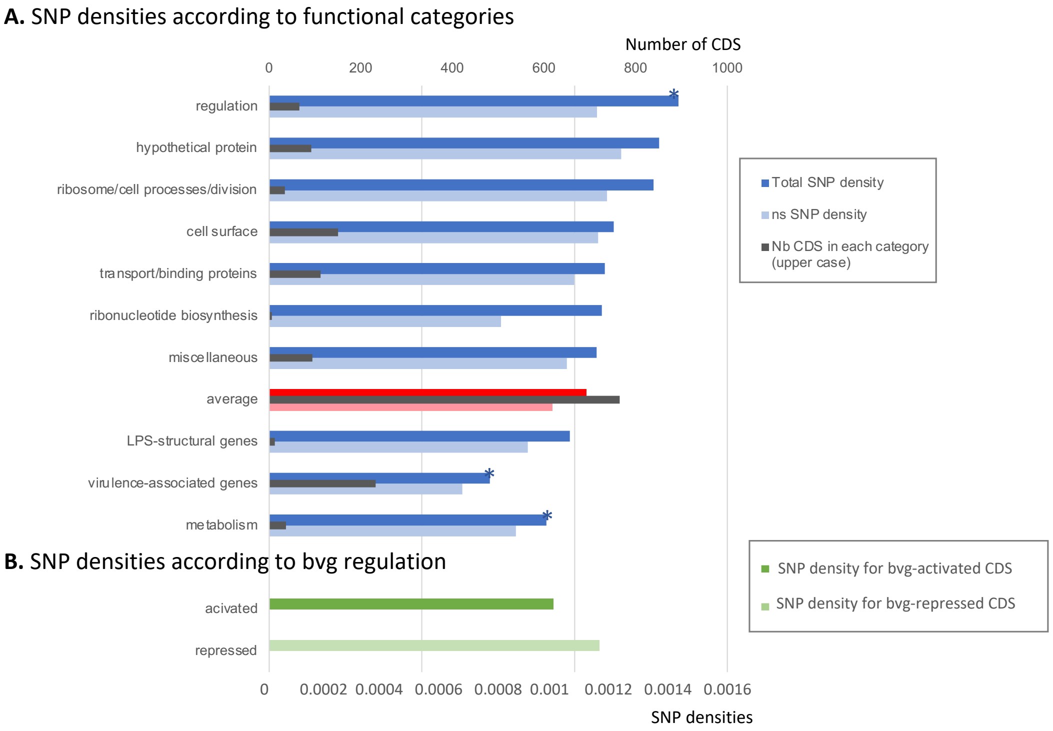

Figure S5. SNP densities according to functional categories (panel A) and to bvg-regulation (panel B). For each functional category: Total SNP densities: blue bars; non-synonymous (ns) SNP densities: light blue; Number of CDS: black bars; Total SNP density in bvg-activated CDS: dark green; and in bvg-repressed CDS: light green. The average (red) is defined as the SNP density of all intragenic SNPs with a known functional category. * indicates $p<0.05$ using a Fisher exact test for each category vs the average.

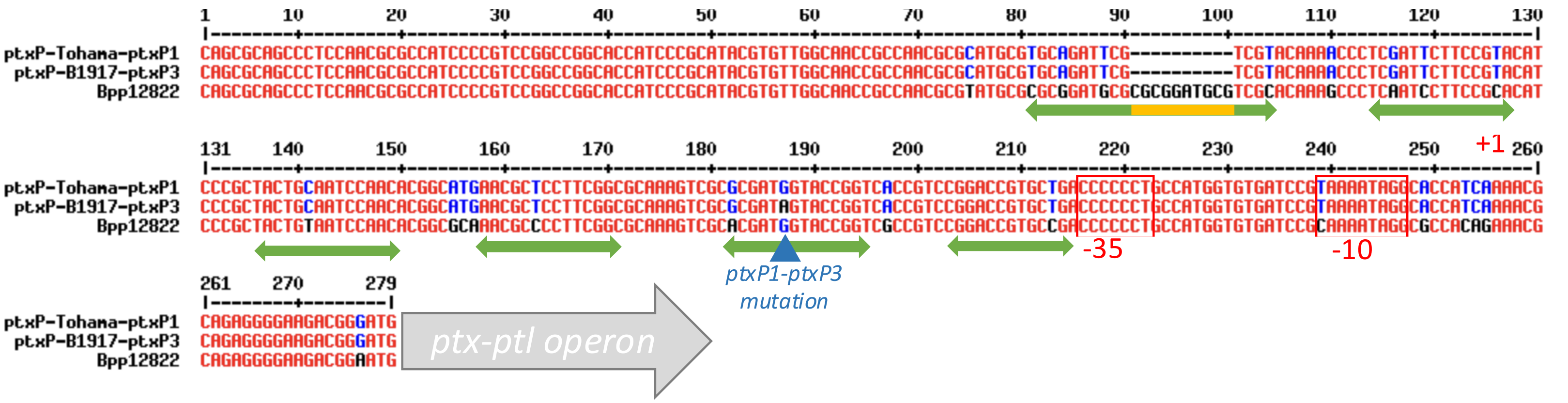

Figure S6: *ptxP* promoter sequence in *Bordetella parapertussis*

Alignment of *ptxP* sequences from Bp (*ptxP1* Tohama strain and *ptxP3* B1917 strain) with the *ptxP* sequence of Bpp strain 12822. Note that the *Bpp ptx*P promoter sequence is 10 bp longer due to a single insertion (CGCGGGATGCG), and has 23 mutations compared to the ptxP1 Tohama sequence.

The 6 green double arrows represent the 6 BvgA ~ P dimer binding sites. The yellow bar that interrupts one of these represents the single insertion (CGCGGGATGCG) within Bpp's ptxP sequence, compared to Bp ptxP sequences. Mutations sites are labelled in blue (when found in 2 sequences) or in black (when found in only 1 sequence). The blue triangle indicates the position that differs between Bp-ptxP1(G) and Bp-ptxP3 (A) alleles. At this position, the ptxP sequence in Bpp has a G, as in the ptxP1 sequence.

342

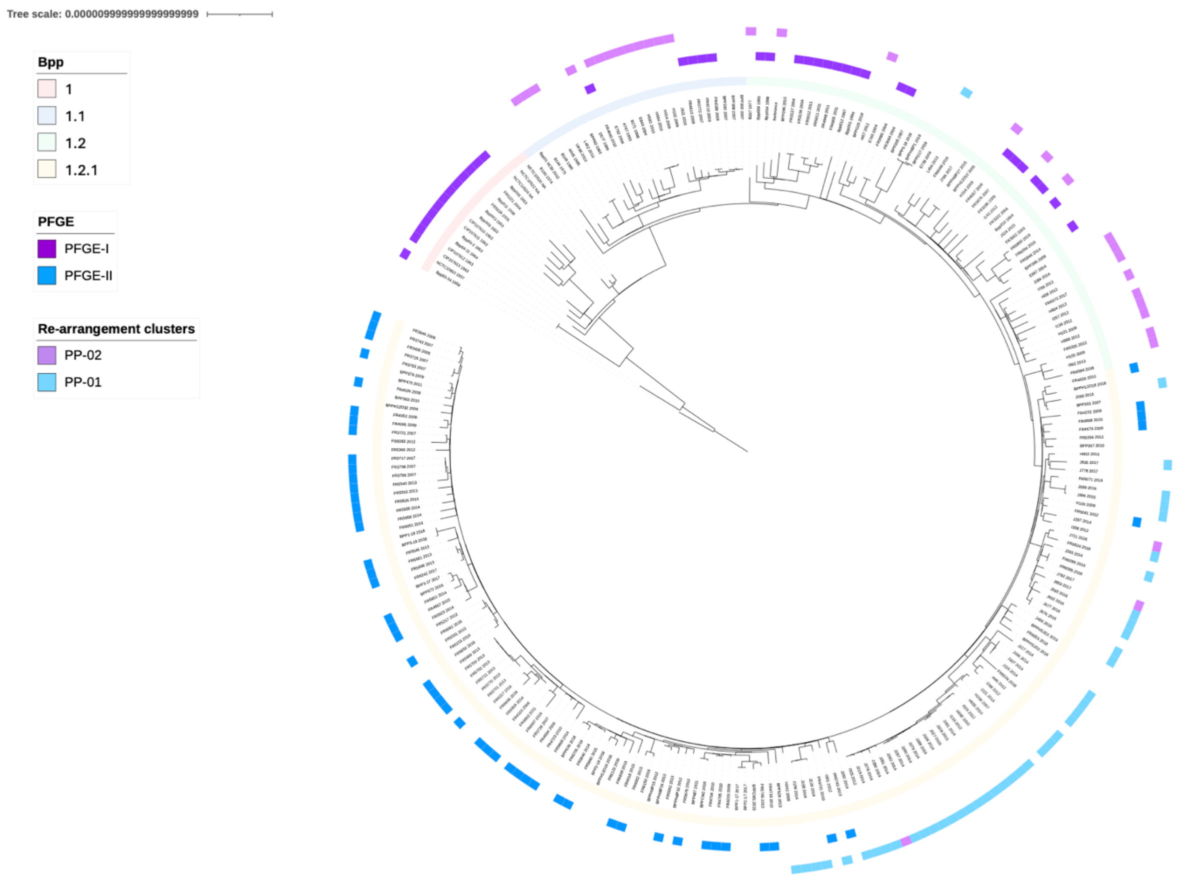

343 **Figure S7. Correspondence between PFGE groups and phylogenetic groups in**
344 ***Bordetella parapertussis*.**
345 Circular phylogenetic tree based on SNPs for the 250 isolates of the study, rooted on Bpp63.34
346 isolate, obtained with Snippy v. 4.3.6 and IQ-TREE version 1.6.10, and visualized in iTOL.
347 The inner circle indicates Bpp phylogenetic groups colored as in **Fig. 2**. The intermediate circle
348 indicates PFGE groups I (purple) and II (blue), which were determined for isolates from France.
349 The outer circle indicates the genomic clusters PP-01 (blue) and PP-02 (purple) as defined in
350 Weigand et al. 2019 [24] for isolates from the USA.
351

Supplementary References

1. Bouchez V, Brun D, Dore G, Njamkepo E, Guiso N. Bordetella parapertussis isolates not expressing pertactin circulating in France. Clin Microbiol Infect **2011**; 17:675–682.
2. Barkoff A-M, Guiso N, Guillot S, et al. A rapid ELISA-based method for screening Bordetella pertussis strain production of antigens included in current acellular pertussis vaccines. J Immunol Methods **2014**; 408:142–148.
3. Bouchez V, Guglielmini J, Dazas M, et al. Genomic Sequencing of Bordetella pertussis for Epidemiology and Global Surveillance of Whooping Cough. Emerg Infect Dis **2018**; 24:988–994.
4. Weigand MR, Peng Y, Loparev V, et al. The History of Bordetella pertussis Genome Evolution Includes Structural Rearrangement. J Bacteriol **2017**; 199.
5. Bowden KE, Weigand MR, Peng Y, et al. Genome Structural Diversity among 31 Bordetella pertussis Isolates from Two Recent U.S. Whooping Cough Statewide Epidemics. mSphere **2016**; 1.
6. Suchard MA, Lemey P, Baele G, Ayres DL, Drummond AJ, Rambaut A. Bayesian phylogenetic and phylodynamic data integration using BEAST 1.10. Virus Evol **2018**; 4:vey016.
7. R Core Team (2021). R: A Language and Environment for Statistical Computing. R Foundation for Statistical Computing, Vienna. 2021; Available at: https://www.R-project.org.
8. Hadley Wickham, Danielle Navarro, and Thomas Lin Pedersen. ggplot2: Elegant Graphics for Data Analysis (3e). Springer, 2016.
9. Bart MJ, Harris SR, Advani A, et al. Global population structure and evolution of Bordetella pertussis and their relationship with vaccination. MBio **2014**; 5:e01074.
10. Moon K, Bonocora RP, Kim DD, et al. The BvgAS Regulon of Bordetella pertussis. mBio **2017**; 8.
11. Cummings CA, Brinig MM, Lepp PW, van de Pas S, Relman DA. Bordetella species are distinguished by patterns of substantial gene loss and host adaptation. J Bacteriol **2004**; 186:1484–1492.
12. Chen Q, Gray MC, Hewlett E, Stibitz S. Four single-basepair mutations in the ptx promoter of Bordetella bronchiseptica are sufficient to activate the expression of pertussis toxin. Sci Rep **2021**; 11:9373.
13. Mooi FR, van Loo IHM, van Gent M, et al. Bordetella pertussis strains with increased toxin production associated with pertussis resurgence. Emerg Infect Dis **2009**; 15:1206–1213.
14. Walker KE, Weiss AA. Characterization of the dermonecrotic toxin in members of the genus Bordetella. Infect Immun **1994**; 62:3817–3828.
15. Linz B, Ma L, Rivera I, Harvill ET. Genotypic and phenotypic adaptation of pathogens: lesson from the genus Bordetella. Curr Opin Infect Dis **2019**; 32:223–230.
16. Brockmeier SL, Register KB, Magyar T, Lax AJ, Pullinger GD, Kunkle RA. Role of the dermonecrotic toxin of Bordetella bronchiseptica in the pathogenesis of respiratory disease in swine. Infect Immun **2002**; 70:481–490.
17. Teruya S, Hiramatsu Y, Nakamura K, et al. Bordetella Dermonecrotic Toxin Is a Neurotropic Virulence Factor That Uses Ca(V)3.1 as the Cell Surface Receptor. mBio **2020**; 11.
18. Jacob-Dubuisson F, Kehoe B, Willery E, Reveneau N, Locht C, Relman DA. Molecular characterization of Bordetella bronchiseptica filamentous haemagglutinin and its secretion machinery. Microbiology (Reading) **2000**; 146 (Pt 5):1211–1221.
19. Lefrancq N, Bouchez V, Fernandes N, et al. Global spatial dynamics and vaccine-induced fitness changes of Bordetella pertussis. Sci Transl Med **2022**; 14:eabn3253.

20. Scheller EV, Cotter PA. Bordetella filamentous hemagglutinin and fimbriae: critical adhesins with unrealized vaccine potential. Pathog Dis **2015**; 73:ftv079.
21. Julio SM, Cotter PA. Characterization of the filamentous hemagglutinin-like protein FhaS in Bordetella bronchiseptica. Infect Immun **2005**; 73:4960–4971.
22. Preston A, Parkhill J, Maskell DJ. The bordetellae: lessons from genomics. Nat Rev Microbiol **2004**; 2:379–390.
23. Parkhill J, Sebaihia M, Preston A, et al. Comparative analysis of the genome sequences of Bordetella pertussis, Bordetella parapertussis and Bordetella bronchiseptica. Nat Genet **2003**; 35:32–40.
24. Weigand MR, Peng Y, Batra D, et al. Conserved Patterns of Symmetric Inversion in the Genome Evolution of Bordetella Respiratory Pathogens. mSystems **2019**; 4.
25. Mäkinen J, Mertsola J, Soini H, et al. PFGE and pertactin gene sequencing suggest limited genetic variability within the Finnish Bordetella parapertussis population. J Med Microbiol **2003**; 52:1059–1063.
